# Maternal and infant outcomes during the COVID-19 pandemic: a retrospective study in Guangzhou, China

**DOI:** 10.1186/s12958-021-00807-z

**Published:** 2021-08-17

**Authors:** Jingyun Wang, Yun Wang, Mei-yao He, Yi-xiao Li, Xin Cheng, Xuesong Yang, Rui-man Li, Guang Wang

**Affiliations:** 1 The First Affiliate Hospital of Jinan University, Jinan University, Guangzhou, 510630 China; 2 International Joint Laboratory for Embryonic Development & Prenatal Medicine, Division of Histology and Embryology, Medical College, Jinan University, Guangzhou, 510632 China; 3 Key Laboratory for Regenerative Medicine of the Ministry of Education, Jinan University, Guangzhou, 510632 China; 4 Guangdong-Hong Kong Metabolism & Reproduction Joint Laboratory, Medical College, Jinan University, Guangzhou, 510632 China

## Abstract

In late December 2019, the COVID-19 pandemic caused a great threat to people’s lives worldwide. As a special category of the population, pregnant women are vulnerable during emergencies. This study was designed to explore whether or not the COVID-19 pandemic has influenced maternal and infant outcomes. We collected maternal characteristics, laboratory results, condition in the third trimester, maternal outcome, fetal or neonatal outcomes, and characteristics of amniotic fluid, umbilical cord and placenta from pregnant women and fetals or newborns in the first affiliated hospital of Jinan university from 24 January to 31 March 2020 (peak period), chose the same types of data at the hospital during the same period in 2019 and 1 January—23 January 2020 (prior to the outbreak of COVID-19 in 2020) as a control. Our study focused on uncomplicated singleton pregnancies among women not infected by COVID-19. The results demonstrated that there was not an increase in adverse outcomes of pregnant women and newborns during the COVID-19 pandemic; This might be associated with the updated design of major epidemic prevention and control systems in Guangzhou, and the extension of pregnant women’s rest time during the third trimester of pregnancy. Nevertheless, the survey showed an increased incidence rate of 25-hydroxyvitamin D and zinc deficiency in newborns during the epidemic, implying that pregnant women should participate in appropriate physical exercise, increase their exposure to outdoor sunlight and improve nutrition intake to ensure healthy newborns during the quarantine period. Our study has provided some guidance for maternal management during the COVID-19 pandemic.

**Supplementary Information:**

The online version contains supplementary material available at 10.1186/s12958-021-00807-z.

## Introduction

In late December 2019, a pneumonia of unknown aetiology unprecedentedly broke out in Wuhan City, Hubei province in China [1]. On 7 January 2020, the novel coronavirus (nCoV) was identified and named Severe Acute Respiratory Syndrome Coronavirus 2 (SARS-CoV-2, COVID-19) [2], infection of which lead to a disease termed COVID-19 by the World Health Organization (WHO) on 11 February 2020 [3]. Most COVID-19 patients have reported mild symptoms, such as dry cough, sore throat, and fever. The majority of cases have spontaneously resolved. However, some have developed severe pneumonia, pulmonary oedema, acute respiratory distress syndrome (aaARDS), or even multiple organ failure and death [4]. On 23 January 2020, the central government of China imposed strict restrictions in and out of Wuhan; shortly thereafter, various restriction measures extended to other cities in Hubei in an effort to control the COVID-19 pandemic [5].

As of 3 July, 2021, the WHO have reported 183,789,535 total COVID-19 cases and 3,973,145 total deaths throughout the world [6]. Among them, there were 118,817 confirmed cases and 5,533 total deaths in China [7]. Both COVID-19 and SARS belong to coronaviruses and can cause severe respiratory infections in people. In 2003, the highest incidence of SARS (12.5 cases per 100,000 people) occurred in Guangzhou city, China [8, 9]. The two earliest recorded COVID-19 cases occurred in Guangzhou on 22 January 2020 [10]. There have been 440 total confirmed cases, 354 discharged cases (15 cases imported overseas) and 1 death in Guangzhou through 31 March 2020 [11].

Since the SARS epidemic and others caused huge negative impacts on population health and socio-economic activities [12], Guangdong province (including Guangzhou city) launched a first-level response to major public health emergencies on 23 January 2020 [13]. Guangzhou's 2020 population is now estimated at over 13 million; it is the third largest city in mainland China and the largest city in the southern part of the country. The population density in Guangzhou city is now approximately 2,000 people per square kilometre or 5,100 people per square mile [14]. Based on pandemic prevention guidelines [15], during the pandemic, non-pharmaceutical interventions included the isolation and separation of sick people with contagious disease from people who are not sick, voluntary quarantine of household members with ill persons, and social distancing measures (e.g., avoiding crowded settings and closing schools and child care centres). However, healthy pregnant women still require both outpatient prenatal care and inpatient delivery services during the COVID-19 pandemic, which shattered the daily routine, social connections and global economy [16].

To date, most COVID-19 pandemic-related studies have focused on potential maternal and infant outcomes from the coronavirus infection [17, 18]. Studies assessing whether or not changes in daily routine, lifestyle and economy affect the health of women during pregnancy without COVID-19 infection and infant development are quite limited. Therefore, we conducted this study to investigate the impact of the pandemic on maternal and infant outcomes in the First Affiliated Hospital of Jinan University during the COVID-19 pandemic (i.e., from the lockdown in Wuhan and launching of the first-level response to major public health emergencies in Guangzhou, China, until now). The goals of this study were to explore whether or not proper psychological or physical interventions affect the health of pregnancy and a baby if the COVID-19 pandemic is enduring.

## Methods

### Participants

The study was conducted with the approval of the Ethics Committee of the First Affiliated Hospital of Jinan University (Guangdong, China). According to the COVID-19 epidemic prevention measures released by the National Health Commission of China, the Guangdong provincial government launched a level-1 response to a major public health emergency on 23 January 2020. The anxiety and panic derived from unpredictable circumstances, quarantine, and sudden travel restrictions during the COVID-19 epidemic affected everyone in Guangzhou. To uncover the impact of the COVID-19 pandemic on maternal and infant outcomes, we collected maternal characteristics, laboratory results, condition in the third trimester, maternal outcome, fetal or neonatal outcomes, and characteristics of amniotic fluid, umbilical cord and placenta from pregnant women and fetals or newborns at the First Affiliated Hospital of Jinan University. In detail, we analysed and compared the data of 1,556 pregnant women and newborns in the First Affiliated Hospital of Jinan University in the early stage 2020 (1 January—23 January 2020), peak period 2020 (24 January—31 March 2020) and the same period of 2019 (24 January—31 March 2019). There were 589 participants in the COVID-19 pandemic outbreak-period group, 733 in the same period of 2019 and 234 in the early stage of the outbreak group.

### Inclusion and exclusion criteria

**Fig. 1:**
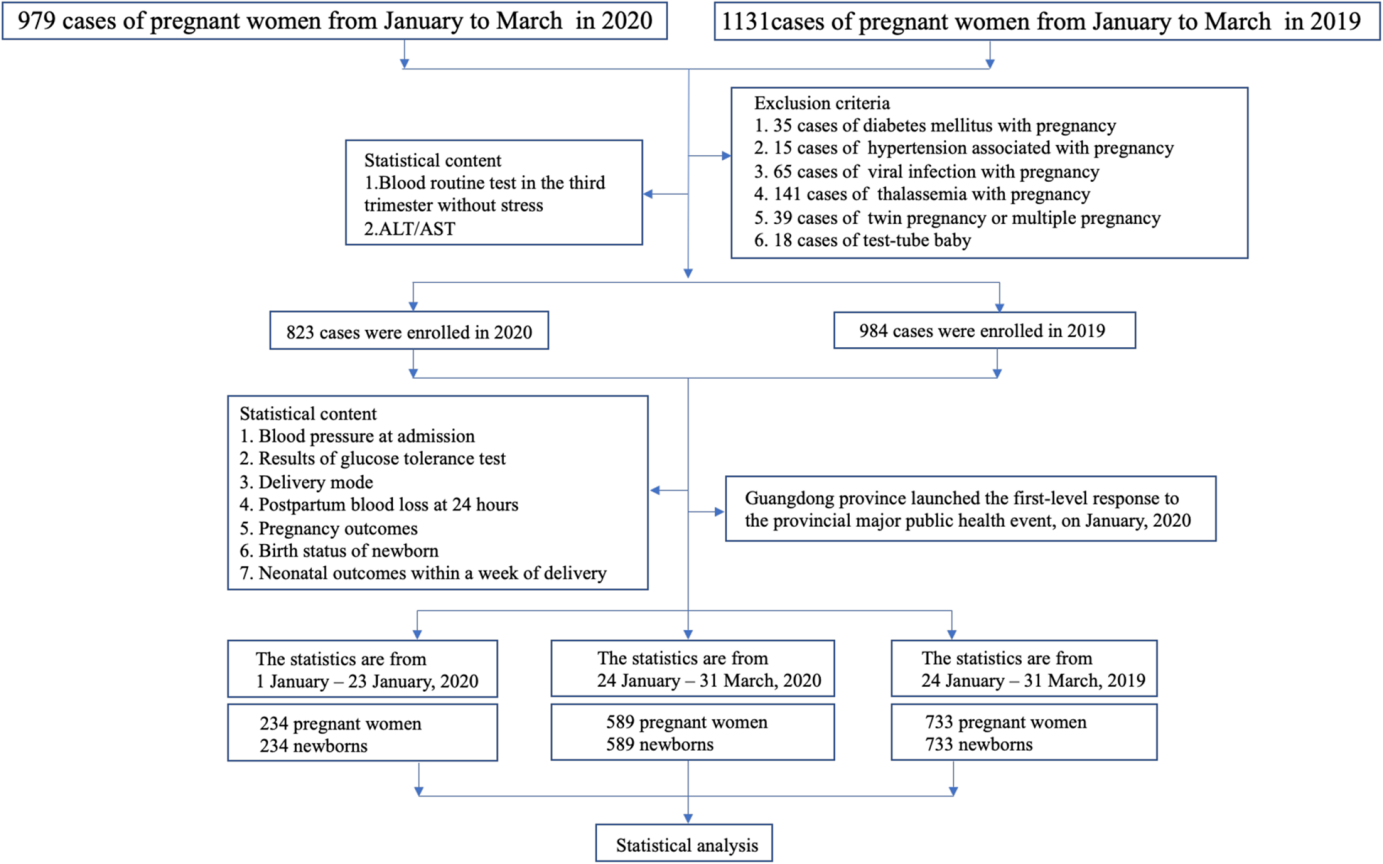
Technical route

Maternal demographic data, labour and delivery information, birth records, and neonatal information were extracted from the First Affiliated Hospital of Jinan University from January to March 2020. As a control, we chose the same types of data at the hospital during the same period in 2019. The labour and delivery information included mode of delivery, gestational age for delivery, postpartum bleeding or PPH, premature rupture of membranes (PROM), placental abruption, placental weight, amniotic fluid volume (AFV) and characteristics, weight and length of newborn, and Apgar score. The laboratory and clinical features of pregnant women included gestational diabetes, obesity and polycystic ovary syndrome (PCOS), hepatitis B, uterine scarring, preeclampsia, placenta previa, vaginitis, group B Streptococcus (GBS), hypothyroidism, upper respiratory tract infections (URTIs), thrombocytopaenia, and intrahepatic cholestasis of pregnancy (ICP). To determine the effect of COVID-19 on circulating levels of blood cytokines in pregnant women, we collected the data of pregnant women during the perinatal period and the clinical laboratory test results in the third trimester of pregnancy without stress, such as the percentages of leucocytes, neutrophils, lymphocytes, haemoglobin, and alanine aminotransferase. According to the birth records for babies born in the hospital, we checked the data of newborn outcomes within one week after birth, such as the number of newborns entering the neonatal intensive care unit (NICU), suffering from infection, shock, septicaemia, asphyxia, newborn respiratory distress syndrome (NRDS), patent foramen ovale (PFO), patent ductus arteriosus (PDA), low birth weight (LBW), giant infant, myocardial injury, neonatal hyperbilirubinaemia, neonatal hypoalbuminaemia, neonatal hypocalcaemia, neonatal vitamin D deficiency, neonatal zinc deficiency, neonatal G6PD deficiency, secondary hair, and major congenital anomalies. The exclusion criteria of pregnant women and their newborns from this study were as follows: diabetes mellitus in pregnancy, hypertension in pregnancy, viral infections during pregnancy, thalassemia, multiple pregnancy, and test-tube baby. The exclusion criteria are shown in detail in Fig. 1.

### Measures

After data collection, 979 pregnant women and newborns were enrolled in the cohort study in 2020, and 1,131 were included in the 2019 cohort. Ultimately, 823 pregnant women and their newborns were enrolled in the cohort study in 2020 and 984 in the 2019 cohort after the exclusion of inappropriate cases. Taking 23 January 2020 as the cut-off point, we first performed statistical analysis on pregnancy outcomes in women between 1 January 2020 and 23 January 2020. Second, the pregnancy outcome data from 24 January 2020 to 31 March 2020 were also analysed statistically. Likewise, we compared the aforementioned data with the previous year’s data of pregnant women and newborns collected from 24 January 2019 to 31 March 2019.

### Statistical analysis

Descriptive statistics were used as a measure of association between the variables; mean and standard deviation were used to measure continuous variables, and frequency and percentage were used to measure categorical variables. Inferential statistics, including independent sample t-test and Pearson's Chi-square test, were used to examine differences in the variables of pregnancy outcomes between the cohorts of 2020 and 2019 over the same period.

## Results

Maternal clinical and laboratory characteristics during the COVID-19 pandemic in the First Affiliated Hospital of Jinan University.

There were no significant differences between the pandemic outbreak-period group and the early-stage outbreak group or the group of the same time frame in 2019 regarding age, body mass index (BMI), blood pressure or numbers of pregnancies and deliveries (Table 1, and Table S1). The oral glucose tolerance test (OGTT) of fasting pregnant women in the pandemic outbreak-period group was higher than in the group of the same time frame in 2019 (*P* = 0.04) (Table 1) and the early-stage outbreak group (*P* = 0.012) (Table S1).

**Table 1:** Maternal Characteristics, According to Study Group

Characteristic	24 January – 31 March 2020	24 January – 31 March 2019	P Value
Median maternal age (Mean ± SD)— yr	30.43 ± 4.13(n = 589)	30.28 ± 4.43(n = 733)	0.63
Median maternal BMI (Mean ± SD) — kg/m^2^	26.58 ± 10.00(n = 588)	26.37 ± 3.18(n = 728)	0.61
Hospital blood pressure— mmHg			
Systolic pressure (Mean ± SD)	119.43 ± 11.21(n = 589)	118.62 ± 11.16(n = 733)	0.07
Diastolic pressure (Mean ± SD)	74.77 ± 8.06(n = 589)	74.00 ± 8.97(n = 733)	0.12
OGTT— mmol/L			
Empty stomach (Mean ± SD)	4.21 ± 0.49(n = 579)	4.16 ± 0.46(n = 709)	0.04*
One hour after meal (Mean ± SD)	7.71 ± 1.76(n = 579)	7.66 ± 2.22(n = 709)	0.34
Two hours after meal (Mean ± SD)	6.70 ± 1.52(n = 579)	6.57 ± 1.48(n = 709)	0.14
Gravidity (Mean ± SD)	2.00 ± 1.12(n = 589)	2.11 ± 1.18(n = 733)	0.12
Number of births (Mean ± SD)	1.52 ± 0.63(n = 589)	1.59 ± 0.65(n = 733)	0.05

Differences between the groups were compared with the Mann–Whitney U test, *p < 0.05, OGTT: Oral glucose tolerance test

Compared with the pandemic outbreak-period group, neutrophil levels (*P* = 0.003) were significantly lower and lymphocytes (*P* = 0.03) and HGB (P = 0.007) were significantly higher than in the group of the same time frame in 2019 (Table 2). Compared with the early-stage outbreak group, the pandemic outbreak-period group showed significantly increased lymphocyte (*P* = 0.005) and haemoglobin (P = 0.01) levels and significantly decreased neutrophils (*P* < 0.001) (Table S2). For the index of liver function, aspartate aminotransferase (AST) levels of pregnant women in the pandemic outbreak-period group were significantly higher than they were in the group of the same time frame in 2019 (*P* = 0.001), whereas alanine aminotransferase (ALT) levels were not significantly different (Table 2). Furthermore, there was no difference in ALT between the pandemic outbreak-period and the early-stage outbreak group (Table S2).

**Table 2:** Maternal Laboratory Results, According to Study Group

Results	24 January – 31 March 2020	24 January – 31 March 2019	P Value
Differential white blood cell count (Mean ± SD) — *10^9/L	8.93 ± 2.08(n = 589)	9.02 ± 2.15(n = 729)	0.41
Neutrophil (Mean ± SD)— no./total no. (%)	70.85 ± 6.27(n = 589)	72.02 ± 6.15(n = 729)	0.003**
Lymphocyte (Mean ± SD)— no./total no. (%)	20.58 ± 5.37(n = 589)	19.95 ± 5.71(n = 729)	0.03*
HGB (Mean ± SD)— g/L	123.31 ± 43.25(n = 589)	120.07 ± 11.49(n = 729)	0.007**
Index of liver function— U/L			
ALT (Mean ± SD)	12.78 ± 11.45(n = 588)	13.35 ± 20.86(n = 725)	0.24
AST (Mean ± SD)	18.65 ± 10.22(n = 588)	17.68 ± 9.29(n = 726)	0.001**

Differences between the groups were compared with the Mann–Whitney U test, *p < 0.05, **p < 0.01, HGB:hemoglobin; ALT:alanine transaminase: AST:aspartate transaminase

To determine maternal characteristics in the third trimester, the incidence rate of preeclampsia and BGS infection significantly decreased in the pandemic outbreak-period group compared with the early-stage outbreak group (Table S3), but there was no significant difference between the pandemic outbreak-period group and the group of the same time frame in 2019 (Table 3). There were no significant differences between the pandemic outbreak-period group and the early-stage outbreak group or the group of the same time (Table 3, and Table S3).

**Table 3:** Maternal Condition in The Third Trimester, According to Study Group

**Maternal condition**	**24 January – 31 March 2020** **(n = 589)**	**24 January – 31 March 2019** **(n = 733)**	**P Value**
Gestational diabetes			
WhiteA1	39/589(6.62)	66/733(9.00)	0.11
WhiteA2	10/589(1.70)	20/733(2.73)	0.21
Pregnancy with obesity	62/589(10.53)	69/733(9.41)	0.50
Hepatitis B	37/589(6.28)	51/733(6.96)	0.63
PCOS	1/589(0.17)	1/733(0.14)	1.00
Scarred uterus	97/589(16.47)	128/733(17.46)	0.63
Uterine fibroids	22/589(3.74)	32/733(4.37)	0.56
Preeclampsia	2/589(0.34)	7/733(0.95)	0.31
Placenta implantation	2/589(0.34)	2/733(0.27)	1.00
Placenta previa	5/589(0.85)	5/733(0.68)	0.73
Vaginitis	18/589(3.06)	26/733(3.55)	0.62
BGS infection	5/589(0.85)	7/733(0.95)	0.84
Hypothyroidism	25/589(4.24)	29/733(3.96)	0.79
Pregnancy with thrombocytopenia	2/589(0.34)	2/733(0.27)	1.00
ICP	11/589(1.87)	12/733(1.64)	0.75

Differences between the groups were compared with the chi-square test or Fisher’s exact test. PCOS: Polycystic ovary syndrome, ICP: Intrahepatic cholestasis of pregnancy

With regard to pregnancy outcomes and complications of pregnant women, the number of pregnant women who had spontaneous vaginal delivery in the pandemic outbreak-period group was significantly higher than that in the group of the same time frame in 2019, among which the number of midwives was also significantly lower than the group of the same time frame in 2019. The number of pregnant women with perineum laceration in the epidemic-peak group was significantly higher than it was in the group of the same time frame in 2019, although there were no differences between the pandemic outbreak-period group and the group of the same time frame in 2019 (Table S4). In addition, there was no difference in the incidence rate of postpartum hemorrhage (PPH), but the amount of bleeding within 24 h in the pandemic outbreak-period group was significantly lower than that in the early-stage outbreak group (*P* = 0.001) (Table S4) and in the group of the same time frame in 2019 (*P* = 0.001) (Table 4).

**Table 4:** Maternal Outcomes, According to Study Group

Outcomes	24 January – 31 March 2020 (n = 589)	24 January – 31 March 2019 (n = 733)	P Value
Method of delivery — no./total no. (%)			
Vaginal birth			
Spontaneous	404/589 (68.59)	442/733 (60.30)	0.002**
Instrument-assisted	15/589 (2.55)	51/733 (6.96)	< 0.001***
Cesarean section	170/589 (28.86)	240/733 (32.74)	0.13
Placental abruption	9/589(1.53)	10/733(1.36)	0.80
Fetal distress	46/589(7.81)	68/733(9.28)	0.34
Precipitate labour	4/589(0.68)	3/733(0.41)	0.71
PROM	158/589(26.83)	186/733(25.38)	0.55
Perineal laceration	336/419(87.35)	337/493(68.36)	< 0.001***
PPH	25/589(4.24)	43/733(5.87)	0.18
24 h (Mean ± SD)—ml	351.97 ± 193.18(n = 589)	367.15 ± 161.53(n = 732)	0.001**

Differences between the groups were compared with the Mann–Whitney U test, and the chi-square test or Fisher’s exact test. **p < 0.01, ***p < 0.001. PPH: postpartum hemorrhage, PROM: premature rupture of membrane

Infant outcomes during the COVID-19 pandemic in the First Affiliated Hospital of Jinan University.

There were no significant differences between the pandemic outbreak-period group and the early-stage outbreak group or the group of the same time frame in 2019 in terms of birth weight and length, the incidence rate of low birth weight, or the incidence rates of adverse foetal development (Table 5, and Table S5). The median gestational age at delivery were larger in the pandemic outbreak-period group than the group of the same time frame in 2019 (*P* = 0.04), but no significant differences were observed compared to the early-stage outbreak group. Compared to the group of the same time frame in 2019, the incidence rate of neonatal infection was decreased in the pandemic outbreak-period group, although there was no significant difference compared to the early-stage outbreak group. The incidence rates of 25-hydroxyvitamin D deficiency were greater in the pandemic outbreak-period group than the group of the same time frame in 2019 and the outbreak early period group. There were no significant differences in hypoalbuminaemia or neonatal hyperbilirubinaemia between the pandemic outbreak-period group and the group of the same time frame in 2019, but there were significant differences between the pandemic outbreak-period group and the early-stage outbreak group. The incidence of zinc deficiency was greater in the pandemic outbreak-period group than the group of the same time frame in 2019, but there were no significant differences between the pandemic outbreak-period group and the early-stage outbreak group (Table 5, and Table S5).

**Table 5:** Fetal or neonatal Outcomes, According to Study Group

Outcomes	24 January – 31 March 2020	24 January – 31 March 2019	P Value
Median gestational age at delivery (Mean ± SD) — wk	39.26 ± 1.42 (n = 589)	39.08 ± 1.63 (n = 733)	0.04*
Full-term birth — no./total no. (%)	560/589 (95.08)	681/733 (92.91)	0.77
Premature birth — no./total no. (%)	29/589 (4.92)	51/733 (6.96)	0.15
Median birth weight (Mean ± SD) — kg	3.21 ± 0.43 (n = 589)	3.22 ± 1.32 (n = 733)	0.86
Median birth length (Mean ± SD) — cm	49.64 ± 1.81 (n = 589)	49.45 ± 2.15 (n = 733)	0.11
Low birth weight infant	13/589 (2.21)	19/733 (2.60)	0.66
Adverse fetal	7/589 (1.19)	11/733 (1.50)	0.63
Adverse neonatal outcomes — no./total no. (%)			
Admission to NICU	122/589 (20.71)	157/733 (21.42)	0.80
Infection	27/589 (4.58)	59/733 (8.05)	0.02*
Shock	1/589 (0.17)	4/733 (0.55)	0.27
Sepsis	1/589 (0.17)	5/733 (0.68)	0.17
Apgar score < 7 after 1 min	10/589 (1.70)	20/733 (2.73)	0.22
Asphyxia	1/589 (0.17)	1/733 (0.14)	0.88
NRDS	5/589 (0.85)	11/733 (1.50)	0.29
Patent foramen ovale	21/589 (3.57)	44/733 (6.00)	< 0.001***
Patent ductus arteriosus	14/589 (2.38)	17/733 (2.32)	0.95
Macrosomia	11/589 (1.87)	25/733 (3.41)	0.10
Myocardial damage	43/589 (7.30)	60/733 (8.19)	0.58
Neonatal hyperbilirubinemia	66/589 (11.21)	95/733 (13.00)	0.39
Hypoalbuminemia	18/589 (3.06)	33/733 (4.5)	0.19
Hypocalcemia	11/589 (1.87)	24/733 (3.27)	0.12
25-hydroxyvitamin D deficiency	16/589 (2.72)	1/733 (0.14)	< 0.001***
Zinc deficiency	46/589 (7.81)	20/733 (2.73)	< 0.001***
Neonatal polycythemia	8/589 (1.36)	3/733 (0.41)	0.06
G6PD deficiency	3/589 (0.51)	2/733 (0.27)	0.49
Caput succedaneum	27/589 (4.58)	26/733 (3.55)	0.36

Differences between the groups were assessed with the use of the Mann–Whitney U test for gestational age; the use of the t test for full-term birth and premature birth; the use of the chi-square test for other test index. *p < 0.05, ***p < 0.001. NICU: neonatal intensive care unit; NRDS: neonatal respiratory distress syndrome; G6PD: Glucose 6-phosphate dehydrogenase

There were no significant differences in umbilical cord length, placental weight, or volume and properties of amniotic fluid between the pandemic outbreak-period group and contemporaneous group or the early-stage outbreak group (Table 6, and Table S6).

**Table 6:** Characteristics of Amniotic Fluid, Umbilical Cord and Placenta, According to Study Group

Characteristics	24 January – 31 March 2020	24 January – 31 March 2019	P Value
Amniotic fluid properties— no./total no. (%)			
Normal	478/588 (81.29)	593/729 (81.34)	0.99
Meconium-stained	101/588 (17.18)	128/729 (17.56)	0.88
Bloody	9/588 (1.53)	8/729 (1.10)	0.49
Median umbilical cord length (Mean ± SD) — cm	50.31 ± 7.89 (n = 587)	50.34 ± 8.12 (n = 732)	0.77
Median Placental weight (Mean ± SD) — g	547.15 ± 90.83 (n = 589)	545.76 ± 90.91 (n = 733)	0.86
Median Amniotic fluid volume (Mean ± SD) — ml	505.44 ± 320.08 (n = 581)	509.95 ± 281.01 (n = 730)	0.17

Differences between the groups were assessed with the use of the Mann–Whitney U test for umbilical cord length, placental weight and amniotic fluid volume; the use of the chi- square test for other test index

## Discussion

Defined as women’s health during pregnancy, perinatal health is an important aspect of public health. However, the effects of the COVID-19 pandemic on healthy pregnant women without COVID-19 infection remain unknown. Pregnant women are regarded as a “vulnerable population” because they are more sensitive to environmental factors, including stress, than the general population. However, women may become decreasingly sensitive to the effects of stress as pregnancy advances [19].

The focus of study is only on uncomplicated singleton pregnancies among women not infected by COVID-19. Our results show that the survey results indicate that there were no differences in the basic characteristics of the two groups, suggesting no significant differences in baseline measurements of health. The total number of white blood cells, ALT, and AST are sensitive biochemical indicators, and the concentrations of these indicators slightly increased but varied within the normal range under stressful conditions (Table 2). Although the proportion of neutrophils and lymphocytes in the observed group was lower than that in the control group, we speculate that this difference did not result in adverse outcomes of pregnant women given that the amounts of ALT and AST were within the normal range of deviation, and there was no difference in the number of white blood cells. The haemoglobin level in pregnant women in the observed group was higher than that in the control group, which may be a beneficial result of nutrition intake [20].

Labour and delivery are affected by many factors, such as maternal history, environmental and behavioural factors, as well as socio-demographic factors (i.e., race, age, and marital status) [21, 22]. Accumulating evidence suggests that extrinsic stress is closely associated with terrible birth outcomes [23], broadening the concept of social determinants of a healthy birth. Previous studies have shown that there are controversial conclusions for determining the key factors to maintain healthy birth in response to natural disasters [24–27]. In agreement with the effect of the Gulf of Mexico oil spill on adverse maternal outcomes [27], this survey found that pregnancy complications, including preeclampsia, gestational hypertension, premature rupture of membranes, and preterm delivery, did not significantly increase during the COVID-19 outbreak compared to the control period (Table 3). Interestingly, we observed several health indicators in pregnant women in the research group, such as slightly longer pregnancy, increased rate of spontaneous vaginal delivery, and reduction of PPH and postnatal psychological morbidity (Table 4). There are several possible explanations for this finding. i) People stayed at home in order to minimise the spread of COVID-19. In particular, pregnant women may be particularly sensitive to self-preservation, and they unconsciously reduced the frequency of ongoing prenatal care (FPC) and prolonged the intervals for prenatal visits to minimise infection risk. Consequently, among symptomatic pregnant women, gestational age was often prolonged because women were not admitted to the hospital timely in a timely fashion. Furthermore, the higher rate of vaginal births may have been due to the reduction of preventive, diagnostic and therapeutic medical interventions. ii) The substantial decrease in the number of people going out was accompanied by blood product shortages, leading obstetricians to become more active participants in the prevention and treatment of PPH. Both the reduction in women who received midwifery care and the increase in the number of spontaneous vaginal deliveries resulted in the reduction of PPH. Although the survey results showed that occurrences of perineal laceration increased in the research group compared with the data in the same period in 2019, we did not find that it affected the average amount of PPH, which suggests that this may have been due to superficial lacerations, less bleeding, and suture and effective haemostasis.

Our study showed that the rate of neonatal infections was reduced, which seems to be associated with the health actions of pregnant women during delivery. Although the survey showed a decrease in the proportion of patent foramen ovale, we did not consider this to be an abnormal indicator, since it is a common phenomenon even in healthy newborns, most of which close spontaneously 7 days postpartum or even after giving birth [28, 29]. Therefore, we could not classify the observation in newborns as congenital heart disease.

Our study showed an increase in the prevalence of 25-hydroxyvitamin D deficiency and zinc deficiency, important elements of foetal growth and development, in newborns in the research group. As an important nutrient in bone health, vitamin D inadequacy may increase the risk of fractures, rickets, osteomalacia, and osteoporosis. Because humans generate vitamin D with the help of sunlight [30], seasonal changes in addition to the use of dietary supplements can also influence the intake of vitamin D [31–33]. The growth and development of the foetus in the uterus are completely dependent on the mother; thus, the storage of vitamin D during pregnancy plays an important role in foetal growth [34]. It has been shown that vitamin D deficiency in pregnant women occurs more frequently in winter than in summer [35]. Hence, we speculate that the home quarantine measures during the COVID-19 pandemic reduced the sunlight exposure of pregnant women, causing maternal vitamin D deficiency and eventually increasing the prevalence of 25-hydroxyvitamin D deficiency in the newborns.

Since these trace elements in newborn babies mainly come from the mother, low serum zinc levels during pregnancy can easily lead to zinc deficiency in infants. Regarding the increased prevalence of newborns’ zinc deficiency in the research group, it is speculated that changes in pregnant women’s dietary structure during the COVID-19 epidemic would interfere with the mean intakes of mineral and trace elements from food. However, this retrospective study provides very limited support for this theory because it failed to monitor the levels of trace elements and vitamins in pregnant women in the research and control groups. For that reason, more rigorous studies are certainly required to reveal the underlying mechanisms involved in neonatal vitamin D and zinc deficiency.

To further verify the impact of the COVID-19 epidemic on maternal and newborn outcomes, we compared one more set of data of pregnant women and newborns as another control group, which was taken from the period (i.e., from 1 January 2020 to 23 January 2020) prior to outbreak of COVID-19 (Table S1, S2, S3, S4, S5 and S6). There was no difference in the health indicators of pregnant women between the research and control groups. Compared to this control group, both the haemoglobin levels in pregnant women and PPH in the research group were comparatively improved. It is worth noting that the incidences of both neonatal zinc and 25 hydroxyvitamin D deficiencies in the research group were higher than in this control group, while the incidence of patent foramen ovale in the research group was lower. However, inconsistent with the previous comparison, we observed a lower incidence of preeclampsia and upper respiratory tract infections, premature delivery, low birth weight, patent ductus arteriosus, and hyperbilirubinaemia and hypoproteinaemia. The comparison of the two groups further confirmed that there were no adverse outcomes of pregnant women or newborns during the COVID-19 epidemic; the outcome of newborns during COVID-19 seemed to be better than those in the absence of COVID-19. In contrast, the outcomes seemed better from the view of the newborn during COVID-19 compared the data prior to the pandemic.

In conclusion, this study revealed that the quarantine measures during the COVID-19 epidemic did not substantially negatively affect maternal or neonate outcomes, which may have been associated with the previous SARS epidemic in Guangzhou. Moreover,improvements in the quality of rest at home as a result of quarantine measures during the COVID-19 epidemic might have yielded more benefits to maternal and infant outcomes. Nevertheless, the vitamin D and zinc deficiencies identified in newborn during the COVID-19 epidemic should draw attention.

## Supplementary Information

**Additional file 1: Table S1.** Maternal Characteristics, According to Study Group.

**Additional file 2: Table S2.** Maternal Laboratory Results, According to Study Group.

**Additional file 3: Table S3.** Maternal Condition in the Third Trimester, According to Study Group.

**Additional file 4: Table S4.** Maternal Outcomes, According to Study Group.

**Additional file 5: Table S5.** Fetal or neonatal Outcomes, According to Study Group.

**Additional file 6: Table S6.** Characteristics of Amniotic fluid, Umbilical Cord and Placenta, According to Study Group.

## Data Availability

The data sets supporting the results of this article are included within the article and its additional files.
