# Supplementary material for: Maternal and infant outcomes during the COVID-19 pandemic: a retrospective study in Guangzhou, China
Source: Reprod Biol Endocrinol. 2021 Aug 17;19:126. doi: 10.1186/s12958-021-00807-z (PMC8369138; doi:10.1186/s12958-021-00807-z)
Supplement: Supplementary file 1 — Additional file 1: Table S1. Maternal Characteristics, According to Study Group. [file 12958_2021_807_MOESM1_ESM.docx]

| **Table S1. Maternal Characteristics, According to Study Group.** | | | |
| --- | --- | --- | --- |
| **Characteristic** | **24 January – 31 March 2020** | **1 January – 23 January 2020** | **P Value** |
| Median maternal age ((Mean ± SD）— yr | 30.43±4.13（n=589） | 30.42±4.48（n=234） | 0.79 |
| Median maternal BMI (Mean ± SD) — kg/m2 | 26.58±10.00（n=588） | 26.59±3.97（n=231） | 0.37 |
| Hospital blood pressure— mmHg | | | |
| Systolic pressure (Mean ± SD） | 119.43±11.21（n=589） | 118.65±14.97（n=234） | 0.09 |
| Diastolic pressure (Mean ± SD） | 74.77±8.06（n=589） | 74.20±9.58（n=234） | 0.17 |
| OGTT— mmol/L | | | |
| Empty stomach (Mean ± SD） | 4.21±0.49（n=579） | 4.14±0.51（n=227） | 0.012* |
| One hour after meal (Mean ± SD） | 7.71±1.76（n=579） | 7.70±1.75（n=227） | 0.86 |
| Two hours after meal (Mean ± SD） | 6.70±1.52（n=579） | 6.80±1.54（n=227） | 0.24 |
| Gravidity (Mean ± SD) | 2.00±1.12（n=589） | 2.10±1.18（n=234） | 0.31 |
| Number of births (Mean ± SD ) | 1.52±0.63（n=589） | 1.54±0.68（n=234） | 0.85 |

Differences between the groups were compared with the Mann–Whitney U test, *p＜0.05，**p＜0.01，***p＜0.001. OGTT： Oral glucose tolerance test.
