# Supplementary material for: Maternal and infant outcomes during the COVID-19 pandemic: a retrospective study in Guangzhou, China
Source: Reprod Biol Endocrinol. 2021 Aug 17;19:126. doi: 10.1186/s12958-021-00807-z (PMC8369138; doi:10.1186/s12958-021-00807-z)
Supplement: Supplementary file 2 — Additional file 2: Table S2. Maternal Laboratory Results, According to Study Group. [file 12958_2021_807_MOESM2_ESM.docx]

| **Table S2. Maternal Laboratory Results, According to Study Group.** | | |
| --- | --- | --- |
| **Results** | **24 January – 31 March 2020 1 January – 23 January 2020** | **P Value** |
| Differential white blood cell count (Mean ± SD）  — *10^9/L | 8.93±2.08（n=589） 9.19±2.35（n=234） | 0.14 |
| Neutrophil (Mean ± SD）— no./total no. (%) | 70.85±6.27（n=589） 72.51±6.47（n=234） | ＜0.001*** |
| Lymphocyte (Mean ± SD）— no./total no. (%) | 20.58±5.37（n=589） 19.50±5.45（n=234） | 0.005** |
| HGB (Mean ± SD）— g/L | 123.31±43.25（n=589） 119.10±7.25（n=233） | 0.01* |
| Index of liver function— U/L | | |
| ALT (Mean ± SD） | 12.78±11.45（n=588） 12.94±7.25（n=233） | 0.42 |
| AST (Mean ± SD） | 18.65±10.22（n=588） 18.73±6.63（n=233） | 0.86 |

Differences between the groups were compared with the Mann–Whitney U test, *p＜0.05，**p＜0.01，***p＜0.001.
