# Supplementary material for: Maternal and infant outcomes during the COVID-19 pandemic: a retrospective study in Guangzhou, China
Source: Reprod Biol Endocrinol. 2021 Aug 17;19:126. doi: 10.1186/s12958-021-00807-z (PMC8369138; doi:10.1186/s12958-021-00807-z)
Supplement: Supplementary file 3 — Additional file 3: Table S3. Maternal Condition in the Third Trimester, According to Study Group. [file 12958_2021_807_MOESM3_ESM.docx]

| **Table S3. Maternal Condition in the Third Trimester, According to Study Group.** | | | |
| --- | --- | --- | --- |
| **Maternal condition** | **24 January – 31 March 2020**  **（n=589）** | **1 January – 23 January 2020**  **（n=234）** | **P Value** |
| Gestational diabetes | | | |
| WhiteA1 | 39/589(6.62) | 20/234(8.55) | 0.33 |
| WhiteA2 | 10/589(1.70) | 1/234(0.43) | 0.13 |
| Pregnancy with obesity | 62/589(10.53) | 28/234(11.97) | 0.55 |
| Hepatitis B | 37/589(6.28) | 20/234(8.55) | 0.25 |
| PCOS | 1/589(0.17) | 1/234(0.43) | 0.49 |
| Scarred uterus | 97/589(16.47) | 41/234(17.52) | 0.72 |
| Uterine fibroids | 22/589(3.74) | 7/234(2.99) | 0.60 |
| Preeclampsia | 2/589(0.34) | 5/234(2.14) | 0.02* |
| Placenta implantation | 2/589(0.34) | 3/234(1.28) | 0.12 |
| Placenta previa | 5/589(0.85) | 5/234(2.14) | 0.13 |
| Vaginitis | 18/589(3.06) | 9/234(3.85) | 0.57 |
| BGS infection | 5/589(0.85) | 9/234(3.85) | 0.003** |
| Hypothyroidism | 25/589(4.24) | 8/234(3.42) | 0.59 |
| Pregnancy with thrombocytopenia | 2/589(0.34) | 4/234(1.71) | 0.06 |
| ICP | 11/589(1.87) | 4/234(1.71) | 0.88 |

Differences between the groups were compared with the chi-square test or Fisher’s exact test; *p＜0.05，**p＜0.01，***p＜0.001. PCOS: Polycystic ovary syndrome， ICP: Intrahepatic cholestasis of pregnancy
