# Supplementary material for: Maternal and infant outcomes during the COVID-19 pandemic: a retrospective study in Guangzhou, China
Source: Reprod Biol Endocrinol. 2021 Aug 17;19:126. doi: 10.1186/s12958-021-00807-z (PMC8369138; doi:10.1186/s12958-021-00807-z)
Supplement: Supplementary file 4 — Additional file 4: Table S4. Maternal Outcomes, According to Study Group. [file 12958_2021_807_MOESM4_ESM.docx]

| **TableS4. Maternal Outcomes, According to Study Group.** | | | |
| --- | --- | --- | --- |
| **Outcomes** | **24 January – 31 March 2020**  **（n=589）** | **1 January – 23 January 2020**  **（n=234）** | **P Value** |
| Method of delivery — no./total no. (%) | | | |
| Vaginal birth | | | |
| Spontaneous | 404/589 (68.59) | 151/234 (64.53) | 0.26 |
| Instrument-assisted | 15/589 (2.55) | 6/234 (2.56) | 0.99 |
| Cesarean section | 170/589 (28.86) | 77/234 (32.91) | 0.25 |
| Placental abruption | 9/589(1.53) | 5/234(2.14) | 0.54 |
| Fetal distress | 46/589(7.81) | 19/234(8.12) | 0.88 |
| Precipitate labour | 4/589(0.68) | 1/234(0.43) | 0.56 |
| PROM | 158/589(26.83) | 58/234(24.79) | 0.55 |
| Perineal laceration | 336/419(80.91) | 122/157(77.71) | 0.51 |
| PPH | 25/589(4.24) | 11/234(4.70) | 0.77 |
| 24hours (Mean ± SD）—ml | 351.97±193.18 (n=589) | 377.14±170.8（n=234） | 0.001** |

Differences between the groups were compared with the Mann–Whitney U test, and the chi-square test or Fisher’s exact test; *p＜0.05, **p＜0.01, ***p＜0.001. PPH: postpartum hemorrhage, PROM: premature rupture of membrane.
