# Supplementary material for: Maternal and infant outcomes during the COVID-19 pandemic: a retrospective study in Guangzhou, China
Source: Reprod Biol Endocrinol. 2021 Aug 17;19:126. doi: 10.1186/s12958-021-00807-z (PMC8369138; doi:10.1186/s12958-021-00807-z)
Supplement: Supplementary file 5 — Additional file 5: Table S5. Fetal or neonatal Outcomes, According to Study Group. [file 12958_2021_807_MOESM5_ESM.docx]

| **Table S5. Fetal or neonatal Outcomes, According to Study Group.** | | | |
| --- | --- | --- | --- |
| **Outcomes** | **24 January – 31 March 2020** | **1 January – 23 January 2020** | **P Value** |
| Median gestational age at delivery (Mean ± SD) — wk | 39.26 ± 1.42 (n=589) | 39.20±1.59 (n=234) | 0.08 |
| Full-term birth — no./total no. (%) | 560/589 (95.08) | 211/234 (90.17) | 0.64 |
| Premature birth — no./total no. (%) | 29/589 (4.92) | 23/234 (9.83) | 0.02* |
| Median birth weight (Mean ± SD) — kg | 3.21 ± 0.43 (n=589) | 3.17 ± 0.52 (n=234) | 0.36 |
| Median birth length (Mean ± SD) — cm | 49.64 ± 1.81 (n=589) | 49.28 ± 2.52 (n=234) | 0.06 |
| Low birth weight infant | 13/589 (2.21) | 14/234 (5.98) | 0.01* |
| Adverse Fetal | 7/589 (1.19) | 3/234 (1.28) | 0.91 |
| Adverse neonatal outcomes — no./total no. (%) | | | |
| Admission to NICU | 122/589 (20.71) | 65/234 (27.78) | 0.09 |
| Infection | 27/589 (4.58) | 18/234 (7.69) | 0.10 |
| Shock | 1/589 (0.17) | 1/234 (0.43) | 0.50 |
| Sepsis | 1/589 (0.17) | 4/234 (1.71) | 0.01 |
| Apgar score <7 after 1 min | 10/589 (1.70) | 4/234 (1.71) | 1.00 |
| Asphyxia | 1/589 (0.17) | 1/234 (0.43) | 0.50 |
| NRDS | 5/589 (0.85) | 3/234 (1.28) | 0.57 |
| Patent foramen ovale | 21/589 (3.57) | 31/234 (13.25) | <0.001*** |
| Patent ductus arteriosus | 14/589 (2.38) | 14/234 (5.98) | 0.01* |
| Macrosomia | 11/589 (1.87) | 5/234 (2.14) | 0.81 |
| Myocardial damage | 43/589 (7.30) | 16/234 (6.84) | 0.30 |
| Neonatal hyperbilirubinemia | 66/589 (11.21) | 45/234 (19.23) | 0.01* |
| Hypoalbuminemia | 18/589 (3.06) | 17/234 (7.26) | 0.01* |
| Hypocalcemia | 11/589 (1.87) | 1/234 (0.43) | 0.12 |
| 25-hydroxyvitamin D deficiency | 16/589 (2.72) | 1/234 (0.43) | 0.04* |
| Zinc deficiency | 46/589 (7.81) | 15/234 (6.41) | 0.52 |
| Neonatal polycythemia | 8/589 (1.36) | 2/234 (0.85) | 0.56 |
| G6PD deficiency | 3/589 (0.51) | 4/234 (1.71) | 0.09 |
| Caput succedaneum | 27/589 (4.58) | 12/234 (5.13) | 0.75 |

*Differences between the groups were assessed with the use of the Mann–Whitney U test for gestational age; the use of the t test for full-term birth and premature birth; the use of the chi-square test for other test index. *p＜0.05，**p＜0.01，***p＜0.001. NICU neonatal intensive care unit; NA not applicable; NRDS neonatal respiratory distress syndrome; and G6PD Glucose 6‐phosphate dehydrogenase.
