# Supplementary material for: Maternal and infant outcomes during the COVID-19 pandemic: a retrospective study in Guangzhou, China
Source: Reprod Biol Endocrinol. 2021 Aug 17;19:126. doi: 10.1186/s12958-021-00807-z (PMC8369138; doi:10.1186/s12958-021-00807-z)
Supplement: Supplementary file 6 — Additional file 6: Table S6. Characteristics of Amniotic fluid, Umbilical Cord and Placenta, According to Study Group. [file 12958_2021_807_MOESM6_ESM.docx]

| **Table S6. Characteristics of Amniotic fluid, Umbilical Cord and Placenta, According to Study Group.** | | | |
| --- | --- | --- | --- |
| **Characteristics** | **24 January – 31 March 2020** | **1 January – 23 January 2020** | **P Value** |
| Amniotic fluid properties— no./total no. (%) | | | |
| Normal | 478/588 (81.29) | 184/233 (79.00) | 0.8 |
| Meconium-stained | 101/588 (17.18) | 45/233 (19.31) | 0.55 |
| Bloody | 9/588 (1.53) | 4/233 (1.72) | 0.85 |
| Median umbilical cord length (Mean ± SD) — cm | 50.31 ± 7.89 (n=587) | 50.12 ± 8.89 (n=234) | 0.94 |
| Median Placental weight (Mean ± SD) — g | 547.15 ± 90.83 (n=589) | 536.54 ± 93.79 (n=234) | 0.41 |
| Median Amniotic fluid volume (Mean ± SD) — ml | 505.44 ± 320.08 (n=581) | 509.13 ± 319.14 (n=231) | 0.81 |

*Differences between the groups were assessed with the use of the Mann–Whitney U test for umbilical cord length, placental weight and amniotic fluid volume; the use of the chi- square test for other test index. *p＜0.05，**p＜0.01，***p＜0.001.
